# What are the prospects for the hormonal IUD in the public sector? A mixed-method study of the user population in Zambia

**DOI:** 10.1186/s12905-022-01745-7

**Published:** 2022-05-15

**Authors:** Aurélie Brunie, Megan Lydon, Kayla Stankevitz, Namwinga Chintu, Claire Brennan, Kendal Danna, Kate H. Rademacher

**Affiliations:** 1 FHI 360, Washington, DC USA; 2 FHI 360, Durham, NC USA; 3 Society for Family Health, Lusaka, Zambia; 4 Present Address: UNAIDS, Geneva, Switzerland; 5 Present Address: RTI, Research Triangle Park, NC USA; 6 PSI, Washington, DC USA

**Keywords:** Hormonal IUD, Levonorgestrel releasing intrauterine device, Zambia, Family planning, Long-acting, Contraceptive, Menstrual bleeding

## Abstract

**Background:**

The levonorgestrel-releasing intrauterine device (IUD)—also known as the hormonal IUD—is a highly effective contraceptive method that has not been widely available in the public sector in Zambia. Early introduction efforts can provide critical insights into the characteristics of users, reasons for method choice, and experiences getting their method.

**Methods:**

We conducted a survey with 710 public sector clients who received a hormonal IUD, copper IUD, implant or injectable in two provinces of Zambia, and additional in-depth interviews with 29 women. We performed descriptive analyses of survey data and fitted multivariable logistic regression models to assess factors associated with hormonal IUD use. Qualitative interviews were analyzed thematically.

**Results:**

Factors associated with hormonal IUD use included full-time or self-employment (relative to both implant and copper IUD use), as well as being older, wealthier, and partner not being aware of method use (relative to implant use only). Common reasons for choosing long-acting methods were duration, perception that the method was “right for my body,” and convenience. In addition, a portion of hormonal IUD acceptors mentioned effectiveness, potential for discreet use, few or manageable side effects, and treatment for heavy or painful periods. Between 83 and 95% of women said that they were counseled about menstrual changes and/or non-bleeding side effects; however, more hormonal IUD acceptors recalled being counseled on the possibility of experiencing reduced bleeding (88%) than amenorrhea (43%). Qualitative interviews indicate that women seek methods with minimal or tolerable side effects. While most women reported their partner was aware of method use, men may be more consistently involved in the decision to use contraception rather than in the choice of a particular method. Qualitative results show an appreciation of the lifestyle benefits of reduced bleeding (especially lighter bleeding), although amenorrhea can be cause for concern.

**Conclusions:**

Initial efforts to introduce the hormonal IUD can provide valuable learnings that can inform broader method introduction to expand choice and better suit women’s needs in Zambia and elsewhere. Scale-up plans should include emphasis on high quality counseling and demand generation.

**Plain English Summary:**

The government of Zambia is committed to increasing access to high-quality contraception and making more choices available to users. To date, the hormonal IUD, a highly effective, long-lasting contraceptive has not been widely available in the country. A study in pilot introduction settings provided insights into why women chose the methods, their characteristics, and their experiences getting their methods. The 710 women in the study received family planning services in public sector settings in two provinces in Zambia. Women in the study who received a hormonal IUD, copper IUD, implant, or injectable completed a quantitative survey; in-depth interviews were also conducted with 29 women. Results showed common reasons for choosing the long-acting methods (hormonal IUD, copper IUD or implants) were their duration, perception that the method was “right for my body,” and convenience. In addition, some hormonal IUD acceptors indicated that they were attracted to the method’s effectiveness, potential for discreet use, few or manageable side effects, and treatment for heavy or painful periods. Qualitative interviews with women also showed that women want contraceptive methods that lead to minimal or tolerable side effects. Male partners were typically aware of contraceptive use; however, men were less involved with decisions about the particular method women selected. Use of the hormonal IUD can lead to reduced menstrual bleeding, and in the interviews, women indicated that they liked reduced bleeding (especially lighter bleeding), although amenorrhea (paused bleeding) can be cause for concern. The results can help inform broader method introduction.

**Supplementary Information:**

The online version contains supplementary material available at 10.1186/s12905-022-01745-7.

## Background

Availability of a range of contraceptive methods can help ensure that women’s and couples’ family planning desires and needs are met. The levonorgestrel-releasing intrauterine device—also known as the hormonal IUD—is a long-acting reversible contraceptive (LARC) that is highly effective at preventing pregnancy and has important non-contraceptive attributes including as a treatment for heavy menstrual bleeding and potentially as an option to reduce anemia [1–3]. Although the method is popular in the United States and other high-income markets, it has historically not been widely available in low- and middle-income countries (LMICs) including in Zambia, largely due to the high prices of commercially available products [4, 5].

Efforts are underway to increase affordable access to the hormonal IUD and facilitate broader introduction and scale-up through public healthcare systems in sub-Saharan Africa [6, 7]. Recent experience has shown that the hormonal IUD could be an important addition to the contraceptive method mix in the region. A study among postpartum women in Kenya and a trial with women living with HIV in South Africa showed high satisfaction and continuation rates with the hormonal IUD that were comparable with or superior to other LARCs [8, 9]. Qualitative interviews in Ghana, Kenya and Nigeria revealed favorable perceptions of the hormonal IUD among key opinion leaders, providers, and early adopters [10–14]. In 2016, a market assessment explored potential demand for the hormonal IUD in Zambia, and results led the national Family Planning Technical Working group to endorse broader introduction of the method [15]. Subsequently, the Ministry of Health affirmed their desire to scale-up the method in the public sector [16].

Initial efforts to introduce the hormonal IUD in Zambia can provide valuable learnings that can inform broader method introduction in Zambia and other countries. A recent pilot study of hormonal IUD introduction with postpartum and postabortion care clients in four provinces in Zambia found high satisfaction and continuation rates among users, with providers also reporting generally high satisfaction among women trying the method [17]. However, existing evidence on uptake of the hormonal IUD among the general population in public sector settings remains limited. Despite some impressive successes with provision of the copper intrauterine device (IUD) and implants in Zambia [18, 19], use of LARCs has been hampered by challenges on both the supply- and demand-sides [20]. The present research conducted at public sites supported by the Society for Family Health (SFH) aims to generate evidence on the potential user base for the hormonal IUD and on why women opt for this method in comparison to other LARCs. Given the popularity of injectables in Zambia, we also included injectable users to support a broader exploration of differences between women choosing various methods. With the exception of another study conducted in Nigeria [21], this is, to our knowledge, a rare side-to-side examination of users of these four methods in real-world conditions. Specific objectives were to describe women choosing the hormonal IUD and women choosing other methods in terms of their characteristics, reasons for method choice, and experiences getting their method.

## Methods

### Study setting

In 2018, 47.5% of married Zambian women and 34% of all women used a modern contraceptive method. The share of IUD use in the method mix for all women was 1.5%, with 96% of IUDs being sourced from the public sector where all methods are free [22].

Donations of unbranded hormonal IUD products have been made by the International Contraceptive Access Foundation, a public–private partnership between Bayer AG and the Population Council, to support initial hormonal IUD introduction efforts in Zambia and other LMICs [23]. In 2017, Population Services International (PSI), its network member, SFH, and WCG Cares introduced the hormonal IUD using donated products in 21 urban, public sector facilities with high pre-existing copper IUD uptake in Copperbelt and Muchinga provinces [24].

### Design and data collection

We conducted a prospective cohort study of long-acting reversible contraceptive users at the 21 SFH-supported public health facilities to measure method-specific continuation rates and assess women’s satisfaction and experiences with LARCs [25]. The present paper draws on the baseline survey with women ages 16–49 who received the hormonal IUD, copper IUD, implant (any type) or three-month injectable from one of the facilities during the recruitment period and follow-up in-depth interviews (IDIs) with a subset of women. All four methods were available at each facility. Research assistants assigned to each clinic worked with clinic staff to identify eligible women and scheduled an in-person structured baseline interview with eligible clients at the health facility, their homes or another agreed-upon location. All the women who were recruited and consented to be part of the study were interviewed until the target sample size was met. Sample size was calculated to generate 95% confidence intervals with 5% precision for 12-month continuation rates for each LARC in the prospective study. We used available estimates for continuation rates in Zambia [26] with identical assumptions for the copper IUD and the hormonal IUD; we also assumed a 3% intraclass correlation and a 45% loss-to-follow-up rate. No additional power analyses were carried out for comparative analyses at baseline or follow-up. We aimed for 415 surveys with LARC users (161 hormonal IUD, 161 copper IUD and 93 implants). For practical reasons, we set the target number of surveys with injectable users to 50. These were interviewed at baseline only to allow a descriptive comparison to LARC users. The dates at which women received their method were extracted from clinic records for independent verification of eligibility, with a maximum duration of 100 days between the day the method was received and the survey.

We conducted IDIs with a purposive subset of hormonal IUD, copper IUD and implant users selected based on survey responses to ensure variation in terms of prior contraceptive use (first-time vs. past users) and fertility intentions (space vs. limit). Evidence indicates that 80% saturation can be reached within 8 IDIs and 90% saturation within 16 IDIs [27]. We aimed for 12–16 IDIs with hormonal IUD acceptors and 12–16 IDIs with users of other LARCs.

Survey question domains included socio-demographic characteristics, contraceptive use history, sources of information about the hormonal IUD, reasons for method choice, potential interest in future use of the hormonal IUD (as applicable), and experiences accessing and receiving services (Additional file 1). IDI guides covered women’s goals for the next five years, reproductive and contraceptive history, experiences with previous method, reasons for method selection, experiences getting their method, and perceptions of the hormonal IUD. Research assistants residing in the communities collected survey data in local languages using tablets between August and November 2018. Four other research assistants conducted, audio-recorded and transcribed IDIs into English. Women traveling for their interview received 50 Kwacha (USD 2.44) as transport reimbursement. We obtained written consent for the survey, and oral consent for follow-up IDIs. Zambia does not place any age restriction on young people’s access to contraception; hence the ethics committee indicated that parental consent is not a legal requirement for persons ages 16–17 though it may be required under the age of 16. ERES Converge (2018-May-007) and the National Research Authority in Zambia and FHI 360’s Protection of Human Subjects Committee in the United States (1185380) approved the study.

### Analysis methods

We performed quantitative analyses in Stata version 15 to compare the characteristics, reasons for method choice, and experiences obtaining their method of the four participant groups according to method received. We fitted two multivariable logistic regression models to examine the factors associated with uptake of the hormonal IUD relative to the copper IUD and to implants, respectively. Injectable users were not included in the multivariable analysis. We included 12 variables related to sociodemographic characteristics, prior contraceptive use, and partner awareness of method use in all models. We confirmed the absence of collinearity using variance inflation factor values. We used adjusted odds ratios with their 95% confidence intervals and assessed significance at the 5% level to examine associations based on the logistic models.

We used NVivo 12 to organize qualitative data for thematic analysis using a coding scheme based on deductive codes related to the study’s objectives and adjusted through initial reading of transcripts. We divided transcripts between four analysts who ran periodic checks for intercoder agreement on 10% of transcripts. We prepared analytic memos to explore patterns in the data and used Excel matrices to summarize and compare the prevalence of key themes across different participant groups within each analysis.

## Results

710 eligible women (153 hormonal IUD, 168 copper IUD, 286 implants, 103 injectables) completed the survey; we excluded another 87 women because they did not meet inclusion criteria upon verification of clinic records. Oversampling was the result of delays in tallying the numbers of completed surveys due to connectivity issues between the central coordinating team and field-based research assistants.

### Survey results

#### Characteristics

Most women were married, with children (Table 1). Forty-three percent of implant acceptors, 39% of injectable users and 22% of hormonal IUD acceptors were between the ages of 16 and 24, compared to only 10% of copper IUD users. On average, women who chose the hormonal IUD and copper IUD were older, had more children and were more educated than injectable and implant acceptors. More wanted to limit childbearing and more were in the upper urban wealth quintile. The proportion of women reporting full-time or self-employment was highest among hormonal IUD users.

**Table 1:** Participant socio-demographic characteristics

	Hormonal IUD n = 153	Copper IUD n = 168	Implant n = 286	Injectable n = 103
Age (%)				
16–24	34 (22.2)	16 (9.5)	124 (43.4)	40 (38.8)
25–34	76 (49.7)	94 (55.9)	124 (43.4)	46 (44.7)
35–49	43 (28.1)	58 (34.5)	38 (13.3)	17 (16.5)
Mean age (SD)	30.1 (6.4)	32.3 (5.9)	26.7 (6.4)	27.6 (6.1)
Married (%)	115 (77.7)	146 (87.9)	211 (75.1)	77 (76.2)
Parity (%)				
0	10 (6.6)	4 (2.4)	21 (7.4)	2 (1.9)
1–2	54 (35.6)	49 (29.9)	152 (53.4)	57 (55.4)
3–4	63 (41.4)	73 (44.6)	81 (28.4)	33 (32.0)
5+	25 (16.5)	38 (23.2)	31 (10.9)	11 (10.7)
Mean number of children (SD)	3.0 (1.9)	3.4 (1.8)	2.4 (1.7)	2.5 (1.6)
Fertility intentions (%)				
Child in < 2 years/timing undecided	20 (13.1)	17 (10.1)	45 (15.8)	24 (23.3)
Child in 2 + years	38 (25.0)	30 (17.9)	107 (37.7)	40 (38.8)
No more children	45 (29.6)	55 (32.7)	44 (15.5)	17 (16.5)
Undecided about more children	49 (32.2)	66 (39.3)	88 (31.0)	22 (21.4)
Highest education completed (%)				
No schooling or some primary	21 (13.8)	20 (11.7)	44 (15.4)	12 (11.7)
Primary	55 (35.9)	65 (38.7)	136 (47.5)	54 (52.4)
Secondary	58 (37.9)	65 (38.7)	87 (30.4)	34 (33.0)
More than secondary	19 (12.4)	18 (10.7)	19 (6.6)	3 (2.9)
Urban wealth quintile (%)^a^				
Lowest	24 (15.7)	16 (9.6)	29 (10.2)	6 (5.9)
Second	13 (8.5)	10 (6.0)	46 (16.3)	25 (24.5)
Middle	28 (18.3)	32 (19.2)	78 (27.6)	29 (28.4)
Fourth	36 (23.5)	40 (23.9)	78 (27.6)	25 (24.5)
Highest	52 (34.0)	69 (41.3)	52 (18.4)	17 (16.7)
Full-time or self-employed (%)	62 (40.6)	47 (28.0)	58 (20.5)	27 (26.4)

^a^Relative wealth was measured using the equity tool for Zambia [28]. The urban version of the equity tool compares participants to the urban population in Zambia

Over 90% of women had previously used modern contraception (Table 2). Between 32 and 44% of women had ever used an implant, and 14–25% an intrauterine method. Most women reported last using a short-acting method, especially injectables. Compared to implant (46%) and injectable (43%) users, more hormonal IUD (52%) and copper IUD (59%) acceptors indicated using a modern method in the three months prior to receiving their current method.

Just over half of injectable and implant users and 69% of copper IUD acceptors had heard about the hormonal IUD, with providers being the most common source of information. Compared to between 81 and 86% for other methods, 73% of hormonal IUD users indicated their partner was aware they had received their method.

**Table 2:** Contraceptive use history and decision-making

	Hormonal IUD n = 153	Copper IUD n = 168	Implant n = 286	Injectable n = 103
Contraceptive use history^a^				
Ever use of modern contraception (%)	142 (92.8)	166 (98.8)	260 (90.9)	95 (92.2)
Ever use of implants (%)	67 (43.8)	65 (38.7)	96 (33.6)	33 (32.0)
Ever use of intra-uterine method (%)	21 (13.7)	42 (25.0)	42 (14.7)	19 (18.4)
Last modern method used (%)				
Hormonal IUD	5 (3.3)	0 (0.0)	1 (0.3)	0 (0.0)
Copper IUD	4 (2.6)	13 (7.7)	2 (0.7)	1 (1.0)
Implant	29 (19.0)	24 (14.3)	47 (16.4)	14 (13.6)
Injectable	65 (42.5)	69 (41.1)	105 (36.7)	39 (37.9)
Pills	1 (0.7)	3 (1.8)	6 (2.1)	4 (3.9)
Male condoms	20 (13.1)	30 (17.9)	36 (12.6)	19 (18.4)
Standard days method	17 (11.1)	22 (13.1)	39 (13.6)	14 (13.6)
Other^b^	0 (0.0)	4 (2.4)	13 (4.5)	0 (0.0)
Never used modern method	11 (7.2)	2 (1.2)	26 (9.1)	8 (7.8)
Used modern method in three months prior to receiving method (%)	80 (52.3)	99 (58.9)	132 (46.1)	44 (42.7)
Had heard about hormonal IUD at time of survey (%)	N/A	115 (68.5)	149 (52.3)	54 (52.4)
Sources of information about the hormonal IUD^c^ (%)				
Provider during visit for method	111 (72.5)	93 (80.9)	125 (83.9)	37 (68.5)
Provider, other visit or referral	42 (27.4)	23 (20.0)	34 (22.8)	15 (27.8)
Friends/family	42 (27.5)	44 (38.3)	48 (32.2)	8 (14.8)
Community volunteer or IPC agent	16 (10.5)	14 (12.2)	19 (12.8)	9 (16.7)
Interested in using the hormonal IUD at any time in the future^c^ (%)	N/A	97 (85.8)	116 (77.9)	28 (51.9)
Knew prior to visit that they wanted to use method (%)	77 (50.3)	127 (75.6)	237 (82.9)	89 (86.4)
Made decision without being influenced by others (%)	65 (84.4)	111 (87.4)	196 (82.7)	81 (91.0)
Partner aware of method use (%)	109 (72.7)	141 (84.9)	227 (81.4)	86 (86.0)

*IPC* Interpersonal communication

^a^For the purpose of this analysis, modern methods include the hormonal IUD, the copper IUD, implants, injectables, pills, emergency contraception and male and female condoms

^b^Other methods include female condoms and standard days method

^c^Among women who had heard about the hormonal IUD. Multiple responses possible

In the multivariable regression models (Table 3), women who were full-time or self-employed had higher odds of using the hormonal IUD relative to the copper IUD. Older women, women in the upper wealth quintile, and women who were full-time or self-employed had higher odds of using the hormonal IUD relative to implants, whereas women whose partner was aware they had their method inserted had higher odds of implant use. Other associations were not found to be statistically significant.

**Table 3:** Adjusted odds ratio estimates and 95% confidence interval from logistic regression analyses

Characteristic (reference group) ^a^	Hormonal IUD versus Copper IUD (n = 314)	Hormonal IUD versus Implant (n = 431)
Variables	OR (95% CI)	OR (95% CI)
Age	0.96 (0.91–1.01)	**1.06 (1.01–1.11)**
Currently married	0.85 (0.40–1.83)	1.79 (0.91–3.54)
Completed secondary school	0.86 (0.50–1.48)	1.49 (0.91–2.44)
Wealth categories (lowest)		
Middle	0.83 (0.44–1.57)	1.07 (0.62–1.87)
Upper	0.84 (0.42–1.65)	**1.88 (1.02–3.48)**
Full-time or self-employed	**2.38 (1.37–4.16)**	**1.93 (1.18–3.17)**
Parity	1.01 (0.82–1.26)	1.09 (0.89–1.34)
Fertility intentions (no/no more children)		
Undecided about having more children	0.93 (0.48–1.81)	0.86 (0.45–1.65)
More children, in > 2 years	1.26 (0.58–2.75)	0.67 (0.34–1.34)
More children, within 2 years or undecided timing	1.16 (0.44–3.06)	0.86 (0.38–1.94)
Prior use of any IUD or IUD	0.67 (0.35–1.30)	0.62 (0.32–1.20)
Prior experience of increased bleeding	0.60 (0.33–1.08)	0.74 (0.44–1.24)
Prior experience of reduced bleeding or amenorrhea	0.84 (0.50–1.41)	0.97 (0.59–1.60)
Prior experience of bleeding disturbances	1.42 (0.77–2.64)	1.11 (0.63–1.95)
Partner aware of method use	0.60 (0.29–1.22)	**0.35 (0.18–0.68)**

^a^Age and parity are interval variables. Other variables are yes/no binary variables, with no as the reference level or categorical variables with the reference level included in parentheses. For urban wealth quintiles, the reference level combines the three lowest quintiles. Statistically significant values (*p* ≤ 0.05) are bolded

#### Method choice

**Fig. 1:**
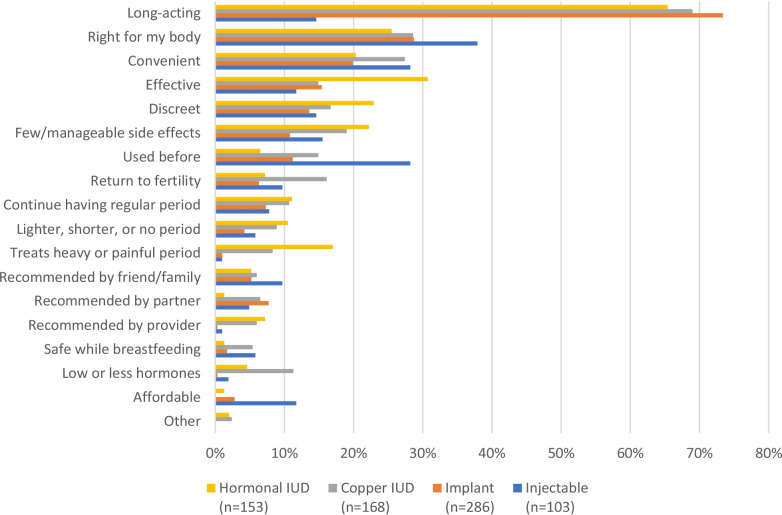
Reasons for method choice. *Multiple responses possible

The most common reason women gave for choosing any of the LARCs was duration (Fig. 1). Other common reasons across all four methods were the method being “right for my body,” and convenience. In addition, over 30% of hormonal IUD acceptors also mentioned effectiveness, discreetness, and few or manageable side effects, while 17% cited treatment of heavy or painful periods. More hormonal IUD and copper IUD users mentioned reduced bleeding or amenorrhea (11% and 9%, respectively) and recommendation by the provider (7% and 6%, respectfully) as a reason for method choice compared to implant and injectable users.

**Fig. 2:**
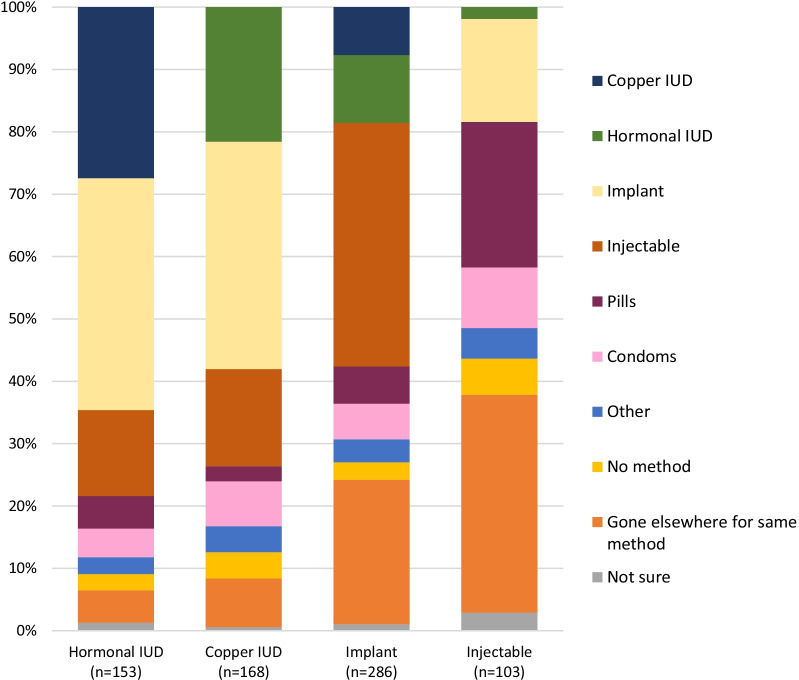
Method participants would have chosen if method received not available. *Gone elsewhere for same method includes implant and injectable users who would have chosen another implant/injectable type

When asked what they would have done if their preferred method was not available, most hormonal IUD and copper IUD users would have selected another LARC (65% and 58% respectively), while most implant and injectable users would have opted for a short-acting method (51% and 68%, respectively) (Fig. 2). More implant (23%) and injectable users (35%) would have gone elsewhere for the same method, compared to hormonal IUD (5%) and copper IUD users (8%). Notably, 22% of copper IUD acceptors and 11% of implant acceptors said they would have chosen the hormonal IUD.

Most women who knew about the hormonal IUD but had received another method said they may be interested in using the hormonal IUD in the future, including 52% of the injectable group, 78% of the implant group and 86% of the copper IUD group (Table 2). Forty-four of the 75 women who were not interested in using the hormonal IUD or unsure said they were afraid of the insertion procedure (results not shown).

#### Experiences obtaining methods

Over 90% of women said they were told about other methods at the time of service provision, and between 83 and 95% said that they were told about menstrual changes and/or non-bleeding side effects (Table 4). When women in the latter group were asked the topics they were counseled on, the proportion of women who mentioned being counseled on at least one type of menstrual change far exceeded that of women who reported being counseled on at least one non-bleeding side effect (97–100% vs. 37–52%). Almost twice as many hormonal IUD acceptors recalled being counseled on the possibility of experiencing reduced bleeding (88%) than amenorrhea (43%), while 74% of copper IUD acceptors said they were informed of possible reduced bleeding but only 30% were informed about possible heavier or prolonged bleeding.

**Table 4:** Experiences with counseling and services, by method received

	Hormonal IUD n = 153	Copper IUD n = 168	Implant n = 286	Injectable n = 103
Told by provider about other methods (%)	143 (93.5)	162 (96.4)	256 (89.5)	95 (92.2)
Told about bleeding changes and/or side effects (%)	146 (95.4)	152 (90.5)	253 (88.5)	85 (82.5)
Side effects mentioned by provider (%) ^a^				
Headaches	24 (16.4)	32 (21.1)	75 (29.6)	33 (38.8)
Nausea/vomiting	9 (6.2)	16 (10.5)	49 (19.4)	23 (27.1)
Weight gain	10 (6.8)	10 (6.6)	58 (22.9)	23 (27.1)
Other	42 (28.8)	62 (40.8)	81 (32.0)	23 (27.1)
*Any type of side effect*	54 (37.0)	66 (43.4)	124 (49.0)	44 (51.8)
Bleeding changes mentioned by provider (%)^a^				
Lighter or shorter bleeding	128 (87.7)	113 (74.3)	136 (53.8)	48 (56.5)
No bleeding	63 (43.2)	53 (34.9)	137 (54.2)	54 (63.5)
Heavier or longer bleeding	34 (23.3)	45 (29.6)	147 (58.1)	56 (65.9)
Bleeding disturbances	65 (44.5)	66 (43.4)	168 (66.4)	51 (60.0)
Less pain during period	36 (24.7)	38 (25.0)	19 (7.5)	7 (8.2)
*Any type of bleeding change*	146 (100.0)	148 (97.4)	250 (98.8)	84 (98.8)
Correctly reported method duration (%)^b^	146 (95.4)	158 (94.0)	260 (92.5)	101 (98.1)
Told by provider at insertion that method can be removed at any time they want (%)	149 (97.4)	165 (98.2)	259 (91.2)	N/A
Told at insertion where removal can be obtained (%)				
Insertion place only	61 (39.9)	31 (18.5)	84 (29.6)	N/A
Place other than insertion place only	2 (1.3)	6 (3.6)	13 (4.6)	N/A
Insertion place and another place	88 (57.5)	126 (75.0)	165 (58.1)	N/A
Not told about any place, don’t know	2 (1.3)	5 (3.0)	22 (7.8)	N/A
Felt privacy sufficient when received method (%)	151 (98.7)	165 (98.2)	284 (99.3)	89 (86.4)
Experienced pain/discomfort when received method (%)^c^	31 (20.3)	28 (16.7)	43 (15.0)	9 (8.7)

^a^Among women counseled on bleeding changes and/or side effects. Multiple responses possible

^b^Based on the duration participants recalled being told by providers when receiving the method. For implants, correct duration was determined based on implant type as informed by participant reports of the number of rods in their implants. Those who did not know their implant type (n = 5) were excluded

^C^Response options included temporary pain, discomfort/pain that lasted a few days, continuing pains, cramping, infection/swelling, scarring or other

Ninety-one percent of implant acceptors and 97–98% of hormonal IUD and copper IUD users said they were told they could remove their method at any time, though between 19 and 40% of LARC users were only told about returning to the place where they had their method inserted as opposed to being given other options for removal.

Almost all LARC acceptors said they had sufficient privacy when they received their method. Between 15 and 20% of LARC acceptors reported experiencing pain or discomfort when they received the method—including temporary pain at insertion, discomfort or pain that lasted a few days and/or scarring.

### IDI results

We completed 29 IDIs, including 16 with hormonal IUD acceptors, two with copper IUD acceptors and 11 with implant acceptors. All IDIs were conducted within 21 weeks of the survey. This section organizes findings according to four themes: women’s motivations for using their method, prior contraceptive experiences, partner’s role, and experiences with service delivery. Table 5 provides a summary of the main factors affecting choice of the hormonal IUD that were identified in the IDIs. Because reduced uterine bleeding is a distinctive feature of the hormonal IUD, interviews also explored women’s general perspectives on reduced bleeding.

**Table 5:** Summary of factors related to the decision to use the hormonal IUD emerging from IDIs

Motivating factors	Demotivating factors
**Shared characteristics with other LARCs** ** Long-acting** ** No resupply/user involvement** ** Reversible** Characteristics of the hormonal IUD Reduced bleeding Therapeutic benefits **Desire to switch methods to avoid menstrual changes/side effects experienced with prior methods** Provider counseling	Little information on method available in the community Perceived novelty of method Fear of intrauterine insertion

Themes that were most prevalent in the IDIs are shown in bold

#### Women’s motivations for using their LARC

When asked about their 5-year goals, most women had economic ambitions, which were often fueled by a desire to better provide for their children. Consequently, many participants reported that LARCs afforded them greater freedom to focus on business or education, while also allowing them to “*rest*” from having children and properly care for the ones they already have. A 23-year-old implant user with a three-month-old baby who aspires to resume her education and become a teacher explained:

[I chose this method] because I knew that after 5 years the baby will be old enough and I would have gone to school…[my husband] said that he was fine with my decision and encouraged me to go ahead with the five-year method to allow the baby to grow before having another child...he wanted me to go to school first as well. – R311

Many women also appreciated the convenience of fewer clinic visits and the greater peace of mind compared to short-acting methods requiring resupply and user involvement. Many participants mentioned the ability to remove the method if they wished to get pregnant or experienced side effects as an important consideration. Some women felt that the 10-to-12-year duration of the copper IUD was too long, although a few acknowledged it could be removed earlier.

A quarter of hormonal IUD users chose the method at least partly because they wanted to reduce or stop their period, in some cases after having experienced increased bleeding on injectables. Several hormonal IUD users also mentioned the method’s therapeutic benefits. A 44-year-old hormonal IUD user with seven children who does not want any more children said:

During counselling I was told that among the benefits of LNG-IUS is not having period. Further I was told that I will not be visiting the clinic frequently to get doses. So, I was happy that this is better because I will not have to come at the clinic regularly and also no buying of sanitary pads meaning [I] am already living like I have reached menopause...They said it happens to prevent cervical cancer. So, I realized that this method was the best, hence I decided to have [it]. – A325

Although several women expressed fear of intrauterine insertions, half of those women had chosen a hormonal IUD or copper IUD. Two hormonal IUD acceptors were initially concerned that the method might fall out during sex or that their partner might feel strings. A couple preferred the hormonal IUD to implants because they feared pain in their arm, and a couple others noted the hormonal IUD was compatible with discreet use. A 39-year-old hormonal IUD acceptor using a LARC for the first time recounted:

[The provider said] that this might be uncomfortable or scary for you since this is your first time getting an insertion in the uterus and I did get scared actually during the procedure… Having something inserted in your uterus it’s not easy, thoughts come like what if something went wrong while inserting, or what if it moves, but later all my fears went because I am used to it now. – A336

#### Prior contraceptive experiences

Across methods, women actively sought contraceptive options with minimal or tolerable side effects and menstrual changes. While many women reported considering information received from close friends, more women indicated not being influenced by what they heard from other women because “*people react differently*.” Notably, there was generally limited discussion of information received about the hormonal IUD from other women in the community. A 47-year-old hormonal IUD user who had previously used pills and injectables said:

When you look at copper T, it has been there for a long time, people know, but with the coming of the LNG-IUS, it’s just been around recently. People can talk but since I heard from the provider, I had full information because most things that are spoken from the community are not correct, especially if something is new, it will always have resistance, but one thing I know is that they cannot bring something that will be harmful to humans. – A331

Many women, most of whom were hormonal IUD acceptors, chose to experiment with a different method out of a desire to avoid bleeding changes or side effects experienced while using prior methods, with several women mentioning prolonged bleeding, stomach pains, headaches and/or weight changes. In several cases, they made this decision after unsuccessful treatment attempts and/or after the provider recommended switching methods. However, women varied in their levels of comfort with trying the hormonal IUD, which they perceived as a new method. Thus, two hormonal IUD acceptors said they wanted to try something new, while a few others admitted to feeling nervous at first. Conversely, a few women opted for the implant because it was more familiar, based on having used it previously or having heard about it in the community. A 31-year-old woman who switched from injectables to the hormonal IUD indicated:

I came here [to the clinic] complaining of headaches and that’s how I was told about the other methods and that’s how I choose this one for 5 years over the one for 12 years…[I chose it] because it has less years than the one for 12 years but they said that it was new… I was scared…We asked if it would bring us problems because it’s new and she said no they are just the same with the 12 years one it’s just the duration that is different. – A321

#### Partner’s role

While women’s partners were often involved in the decision to use contraception, engagement in method choice varied. In addition to alignment on fertility intentions, one consideration mentioned in some interviews was the potential impact on sex life either as an advantage of LARC use over condoms or withdrawal, or as concerns around feeling the strings of the hormonal IUD during sex.

Many women, mostly implant users, said that their husband supported their choice of a LARC method, often for its long duration. Partners of two implant and two hormonal IUD users proposed the method themselves. In some cases, women also reported being supported or encouraged by their partners to try a different method after experiencing side effects or menstrual changes.

In contrast, three hormonal IUD users told their partner about their method only after getting it inserted for reasons including conflicting childbearing intentions, not expecting to change methods when initially reporting to the clinic, and concerns around potential acceptability for fear of interference with sexual intercourse.

#### Service delivery

A few hormonal IUD users reported that provider counseling on the method was a deciding factor. At the same time, several women referred to methods by their duration in their interview and emphasized this characteristic in their choice, with one hormonal IUD user explaining that provider counseling overemphasized duration of effectiveness:

Before getting any contraceptive method, all the methods are discussed. But still, what we just hear is that all of them prevent pregnancies, some being pills to take daily, then there are also injections for 2 months and 3 months and the injection on the arm [implant] for 5 years, and now this same one LNG-IUS. They do not say the medicine in the injections and the only guidance is the duration. Can the medicine also be explained to help us know because of the different bodies women have and the effects we encounter after using the contraceptives? – A322

Most women obtained their method on their first visit. However, about a third of women had to return to the clinic for reasons including stock-outs (for the hormonal IUD), the provider being unavailable, or not coming on a family planning day, with one woman also reporting that upon completing required tests, the clinic hours ended before the method could be inserted.

#### Perspectives on reduced bleeding

IDIs explored women’s perspectives on reduced bleeding and amenorrhea through discussion of prior contraceptive experiences and direct questioning. About half of women expressed negative views of amenorrhea, most of whom believed that periods cleanse the body and flush out “dirt.” Several feared that accumulated blood could cause stomach pain, fibroids or cancer, while a few women said they would worry they were pregnant.

Over a third of women perceived or had experienced benefits of amenorrhea or felt it would not be a concern, while a similar number of participants saw reduced bleeding (mostly discussed as lighter, rather than shorter, bleeding) as a positive characteristic. Many women highlighted financial and lifestyle improvements, primarily buying and using fewer pads, with a few women mentioning greater freedom and confidence and positive impacts on sexual relationships. An 18-year old implant acceptor who is a first-time contraceptive user said:

My flow has become lighter…I was told at the facility that some people experience such side effects so I received it well… sometimes because [of] my heavy flow it was difficult to wear jeans, now even when I am on my period I am still comfortable enough to wear what I want because of the lighter flow and shorter days…[my husband] is happy because it gives us more time to be intimate…even at the farm this has helped me because my energy levels are not so high when I am on my period so with shorter days I work better even through my period days…at least in the extra money for pads I am able to buy a few extra baby clothes for my little girl. – R411

## Discussion

The demographic profile of clients was largely similar across methods, particularly when comparing hormonal IUD and copper IUD users. However, multivariable analyses found that women who worked full-time or were self-employed were more likely to choose the hormonal IUD than other LARCs. Discreet use was also more likely among hormonal IUD users relative to implant users which is a noteworthy finding as clandestine use can be important for family planning users [29]. While most women reported their partner was aware of method use, qualitative results indicate that men may be more consistently involved in the decision to use contraception rather than in the choice of a particular method. Moreover, agreement with partner on method choice often appeared to relate to how long methods protect against pregnancy.

Our analyses indicate that older women and women in the upper wealth quintile were more likely to use the hormonal IUD relative to implants. Importantly, however, almost a third of the study population was between the ages of 16 and 24; a sizeable minority of women in the study also had a lower educational background or were in the lower wealth quintiles across methods. This demonstrates that there is demand for the hormonal IUD across a range of demographics, including traditionally underserved populations such as youth and less educated and poorer women. The public sector, where methods are free, shows potential to reach these women with the hormonal IUD.

Across methods, the large majority of women had prior experience using modern contraceptives, including with LARCs and especially implants. When asked what they would have chosen if their preferred method was not available, implant users would overall have opted for a short-acting method, while the majority of copper IUD and hormonal IUD users would have chosen another LARC. Although courtesy bias should be considered, interest in using the hormonal IUD in the future was also high among both implant and copper IUD users. Quantitative and qualitative findings show that women have a range of reasons for choosing a method. Some of the most common motivations for taking up the hormonal IUD were characteristics shared with other LARCs, including duration of effectiveness and convenience, which women indicated enabled them to pursue their aspirations to improve their lives and those of their children. This suggests that by offering another long-acting option, the hormonal IUD could help women find an option that meets their contraceptive needs and desires.

Side effects are a key cause of discontinuation and method switching in Zambia and across LMICs [22, 30]. As documented elsewhere [31], we found that women try methods, based on their needs and experiences, to find the one that fits them. The distinctive therapeutic benefits of the hormonal IUD for dysmenorrhea and menorrhagia were important for a sizeable minority of women, including some who had experienced these issues because of prior method use. Due to the localized release of hormones, the hormonal IUD causes minimal systemic side effects. Most women also experience a decrease in uterine blood, possibly leading to amenorrhea [5]. Effect on menstruation and non-bleeding side effect profile were both important factors for method choice for some women in survey results, although this was not unique to the hormonal IUD, especially when comparing to reasons reported by copper IUD users. Moreover, while qualitative findings highlight an appreciation of the lifestyle benefits of reduced bleeding (especially lighter bleeding), amenorrhea can be cause for concern for some. This is consistent with findings from other settings, as recently summarized in a systematic review [32].

Quantitative and qualitative results indicate that provider counseling can be a deciding factor in opting for an IUD. Women’s reports of their experiences obtaining methods show that they received important counseling messages including about contraceptive options, potential bleeding changes and non-bleeding side effects with method use, duration of protection and the ability to seek removal if/when desired. Women were also generally satisfied with privacy arrangements and insertion procedures. These results are encouraging in terms of the ability of public sector providers to successfully provide the hormonal IUD in Zambia. However, findings also point at a few areas for improvement in terms of counseling. Since the study was conducted at facilities where the hormonal IUD is available, the fact that slightly over half of implant and injectable users and two-thirds of copper IUD users had not heard about the method was surprising. Another area identified for improvement is counseling messages around bleeding changes and non-bleeding side effects. Though results must be interpreted cautiously since they are based on client self-reports, one important shortcoming was provision of information on the possibility of amenorrhea to hormonal IUD users, with other gaps similarly noted for other methods. This may also explain why some copper IUD users erroneously believed reduced bleeding to be a benefit of the method. Qualitative findings also suggest that providers may sometimes emphasize duration of protection over other product characteristics, with findings further showing evidence of misunderstanding of differences between features of the copper IUD and of the hormonal IUD or of proven therapeutic benefits of the hormonal IUD (for example, while hormonal IUD use reduces the risk of endometrial and ovarian cancers, there are currently insufficient data to draw conclusions for cervical cancer) [1, 33]. Given that contraceptive-induced bleeding changes are particularly well-documented barriers to continuation and amenorrhea has mixed acceptability in many settings, this needs to be addressed; evidence-based counseling may increase the likelihood that users will tolerate bleeding changes, including amenorrhea [33].

Similar to findings from another hormonal IUD pilot program in the region [17], qualitative interviews indicate that some women may not be willing to try a method they are hearing about for the first time. Since the hormonal IUD was relatively new in Zambia, it is not surprising that many women learned about this method from providers. Feedback from other women on their experiences with the hormonal IUD, which can be an important consideration when selecting a method, remains limited. Demand generation, including efforts featuring champion users, should be considered as part of scale-up efforts. Given that findings also indicate that many women acknowledge that methods affect women differently, introduction strategies should always reinforce high quality counseling that emphasizes choice and volunteerism, including a woman’s right to discontinue use of a method at any time.

### Limitations

The generalizability of study findings is limited by the implementation context, which consists of facilities with high copper IUD uptake and that benefit from NGO support. While the study was conducted over a year after the hormonal IUD was introduced in these sites, findings may not be representative of typical public sector settings. The study does not use probability sampling based on a comprehensive sampling frame; as such, results may not be representative of the larger client population. There is also a possibility of selection bias as some women may have been missed by the recruitment approach. The study also did not differentiate between types of implants (one-rod and two-rod).

## Conclusion

The hormonal IUD has the potential to become an important option to increase contraceptive method choice in Zambia. Since this study was conducted, the Ministry of Health in Zambia has decided to introduce the hormonal IUD more broadly in the public sector as part of a comprehensive method mix. To fully realize the potential of the method—both as an option to expand the method mix and potentially to increase overall contraceptive prevalence—introduction and scale-up efforts should include emphasis on high quality counseling as well as demand generation to increase awareness of the method and its distinctive features.

## Supplementary Information

**Additional file 1:** Baseline survey.

## Data Availability

The quantitative datasets and the qualitative topic guides supporting the conclusions of this article are available in the Harvard Dataverse repository, https://dataverse.harvard.edu/dataverse/fhi360_leap.

## Abbreviations

IUD: Intrauterine device
LARC: Long-acting reversible contraceptive
LMIC: Low- and middle-income country
PSI: Population Services International
SFH: Society for Family Health
IDIs: In-depth interviews
USD: U.S. Dollars
