# Supplementary material for: What are the prospects for the hormonal IUD in the public sector? A mixed-method study of the user population in Zambia
Source: BMC Womens Health. 2022 May 15;22:178. doi: 10.1186/s12905-022-01745-7 (PMC9107745; doi:10.1186/s12905-022-01745-7)
Supplement: Supplementary file 1 — Additional file 1: Baseline survey. [file 12905_2022_1745_MOESM1_ESM.docx]

## Identification Questions

| **No.** | **Question** | **Responses** | **Code** | **Skip** |
| --- | --- | --- | --- | --- |
|  | Date of interview  *(automatically recorded on tablet)* | [__\|__] – [__\|__] – [__\|__\|__\|__]  day month year | |  |
|  | Interview start time  *(automatically recorded on tablet)* | [__\|__] : [__\|__]  hrs min | |  |
|  | Interview end time  *(automatically recorded on tablet)* | [__\|__] : [__\|__]  hrs min | |  |
|  | Enter your RA code  ***RA code is a value from 1-25.*** | [__] | |  |
|  | Select the province | Copperbelt  Muchinga | 1  2 |  |
|  | Select the facility | Numeric values representing facilities (labels removed for de-identification) | 1-21 |  |
|  | Enter serial number of participant from the baseline log | [__\|__\|__] | |  |
|  | Select the location of the interview | Participant’s home  Health center  Other location (Specify _____________) | 1  2  3 |  |

## Eligibility and Contact Information

| **No.** | **Question** | **Responses** | **Code** | | **Skip** |
| --- | --- | --- | --- | --- | --- |
|  | Did the participant consent to be interviewed? | Yes  No | 1  0 | | **🡪 END** |
| Before we get started, I need to record your age and a few other questions to help us identify you. | | | | | |
|  | How old were you at your last birthday? | [__\|__] years | | | **If under 16 or over 49, END interview** |
|  | What is your FP number or your NIC number? We will not share this with anyone. This is to help us re-confirm we are talking to the same person when we contact you again for the other surveys? | *A. Enter type of number provided*  FP client number  NIC number  No number | | 1  2  3 |  |
|  |  | *B. Enter number*  *If No number, enter 99.*  [__\|__\|__\|__\|__\|__\|__\|__\|__] | | |  |
|  | What is the name we should use when we contact you again for the other interviews? | _____________________________ | | |  |
|  | What is your address? |  | | |  |
|  | What is the phone number we should use to reach you going forward?  ***Enter 999999999 if phone number unavailable.***  **Probe:** Is there an alternate phone number we can use to reach you? | Main number [__\|__\|__\|__\|__\|__\|__\|__\|__]  Alternate number 1 [__\|__\|__\|__\|__\|__\|__\|__\|__]  Alternate number 2 [__\|__\|__\|__\|__\|__\|__\|__\|__] | | |  |
|  | In addition to the three times we will talk to you like I told you, we would like to contact some women again in a few weeks to ask more questions similar to the questions I will ask you today. This will be more like a conversation to help us better understand their experiences.  May we contact you again in a few weeks for an in-person conversation? | Yes  No | 1  0 | |  |

## Verification Questions

Thank you for sharing that information with us. Before we start this interview, I need to know which contraceptive method you are using.

| **No.** | **Question** | **Responses** | **Code** | **Skip** |
| --- | --- | --- | --- | --- |
|  | Which method did you recently receive? | LNG-IUS  Copper IUD  1-rod implant/Implanon  2-rod implant/Jadelle  Implant - other/unknown  3-month injectable/Depo  Other | 1  2  3  4  5  6  7 | **🡪END** |
|  | Record name of method  Code based on Q016 | LNG-IUS  Copper IUD  Implant  3-month injectable | 1  2  3  4 |  |

## Section 1: Participant Characteristics

| **No.** | **Question** | **Responses** | **Code** | **Skip** |
| --- | --- | --- | --- | --- |
|  | What is the highest level of schooling you completed? | No education / no school  Some primary  Primary  Some secondary  Secondary  Higher  Not sure  No response | 1  2  3  4  5  6  88  99 |  |
|  | What is your religion? | Catholic  Protestant  Muslim  Traditional  No religion  Other  No response | 1  2  3  4  5  6  99 |  |
|  | What is your current work situation?  ***If more than one response applies, record response for primary work*** | Unemployed  Student  Housewife  Casual/part-time employment  Full-time employment: Government civil service  Full-time employment: Private enterprise  Self-employed: Own/family business  Other, specify: __________  No response | 1  2  3  4  5  6  7  8  99 | **🡪Q105**  **🡪Q105**  **🡪Q105**  **🡪Q105**  **🡪Q105** |
|  | What is your primary occupation? | Professional (doctor, lawyer, accountant, lecturer)  Highly skilled (nurse, teacher, School of Tech graduate)  Skilled (tailor, beautician, plumbing, hairdresser, carpentry, electrician)  Semi-skilled (farming, fishing, mining, forestry)  Unskilled (laborer, trader, shopkeeper, hawker, vendor etc.)  Other, specify: _______________  No response | 1  2  3  4  5  6  99 |  |
|  | What is your marital status? | Single  Cohabitating  Married  Divorced / Separated / Widowed  No response | 1  2  3  4  99 | **🡪Q107**  **🡪Q107**  **🡪Q107** |
|  | Is your husband / partner living with you now, or is he staying elsewhere? | Living with participant  Staying elsewhere permanently  Traveling / away but returning  No response | 1  2  3  99 |  |
|  | Have you ever given birth? | Yes  No  No response | 1  0  99 | **🡪Q111**  **🡪Q111** |
|  | When did you last give birth?  ***Accept estimates. Round response to nearest month.***  ***If respondent is unsure, try to use memorable events or age of child if still alive to calculate backwards*** | 0-5 months ago  6-11 months ago  12 or more months ago  No response | 1  2  3  4 | **🡪Q110**  **🡪Q110** |
|  | Have you had a period (menses) since you last gave birth? | Yes  No  No response | 1  0  99 |  |
|  | How many children of your own do you have?  ***If no response enter 99*** | [__\|__] children | |  |
|  | Would you like to have a / another child or would you prefer not to have any / anymore children? | Have a / another child  No / no more children  Undecided  No response | 1  0  88  99 | **🡪Q201**  **🡪Q201**  **🡪Q201** |
|  | How long would you like to wait from now before the birth of a / another child?  ***First choose unit of time, then enter amount.*** | *A. Enter unit of time the participant responds in*  Days  Weeks  Months  Years  Not sure  No response | 1  2  3  4  88  99 |  |
|  |  | *B. Enter amount of time, corresponding to unit previously selected.*  *If Not sure or No response, enter 99.*  [__\|__] | |  |

## Section 2: Contraceptive Use History

Now, let’s talk about contraceptive methods you may have used before you recently received your [name of method] at the clinic.

| **No.** | **Question** | **Responses** | **Code** | | | | **Skip** |
| --- | --- | --- | --- | --- | --- | --- | --- |
|  | Before you received the [name of method] at the clinic, had you ever used an LNG-IUS? | Yes  No  No response | 1  0  99 | | | |  |
|  | Before you received the [name of method] at the clinic, had you ever used a copper IUD? | Yes  No  No response | 1  0  99 | | | |  |
|  | Before you received the [name of method] at the clinic, had you ever used a contraceptive implant? | Yes  No  No response | 1  0  99 | | | |  |
|  |  |  |  | | | |  |
|  | Before you received the [name of method] at the clinic, had you ever used:  **[PLEASE READ EACH RESPONSE OPTION ALOUD]:** | Injectables  Pills  Emergency contraception  Male condoms for contraception  Female condoms for contraception | **Yes**  1  1  1  1  1 | | **No**  0  0  0  0  0 | |  |
|  | Calculated variable, if Q201=0 and Q202=0 and Q203=0 and Q205=0, skip to Q301 |  |  | | | |  |
|  | Did you ever experience changes in your period (menstrual bleeding) while using any of the contraceptive methods you just told me about?  **[ASKING ABOUT BLEEDING CHANGES EXPERIENCED WITH ALL METHODS USED BEFORE THE METHOD INSERTED AT THE CLINIC WHEN THE PARTICIPANT WAS TOLD ABOUT THE STUDY]** | Yes  No  Not sure  No response | 1  0  88  99 | | | | **🡪Q209**  **🡪Q209**  **🡪Q209** |
|  | Which changes in your period (menstrual bleeding) have you experienced while using any of the contraceptive methods you just told me about?  **[ASKING ABOUT BLEEDING CHANGES EXPERIENCED WITH ALL METHODS USED BEFORE THE METHOD INSERTED AT THE CLINIC WHEN THE PARTICIPANT WAS TOLD ABOUT THE STUDY]**  ***Multiple responses possible***  **Probe:** Any other changes in your period (menstrual bleeding)? | Heavier period [a]  Lighter period [b]  Longer period [c]  Shorter period [d]  Bleeding disturbances (periods less or more often, spotting, irregular) [e]  Stopped having period [f]  Other [g]  Not sure [h]  No response [i] | **Yes**  1  1  1  1  1  1  1  1  1 | **No**  0  0  0  0  0  0  0  0  0 | | | **If response to [h] or [i] is 1, no other 1 responses allowed** |
|  | Of the methods you just told me about, which one did you use most recently, before you received your method at the clinic?  ***Looking for information on the method used most recently before the method inserted at the clinic when told about the study.***  ***If participant names a different method that is not on the list, probe about the method used before that until she names one of the response options*** | Copper IUD  LNG-IUS  IUD – unspecified  1-rod implant / Implanon  2-rod implant / Jadelle  Implant – other / unknown  3-month injectable/Depo  2-month injectable/Noristerat  Injectable – other/unknown  Pills  Emergency contraception  Male condom only  Female condom only  SDM / CycleBeads  LAM  Diaphragm  Foam/jelly  No response | 1  2  3  4  5  6  7  8  9  10  11  12  13  14  15  16  17  99 | | | |  |
|  | Why did you stop using that method?  ***Referring to the method used most recently before the method inserted at the clinic when told about for the study.***  ***Multiple responses possible***  **Probe:** Any other reason? | Method reached duration of use [a]  Became pregnant while using [b]  Wanted to become pregnant [c]  Infrequent sex / partner away / lost partner [d]  Partner disapproved [e]  Wanted more effective method [f]  Wanted method lasting longer [g]  Inconvenient / difficult to use [h]  Changes to period [i]  Changes in sex drive [j]  Other side effects [k]  Did not want hormones [l]  Difficulty getting method (too far / too expensive / not available) [m]  Fear of becoming infertile [n]  Other, specify: ___________ [o]  Not sure [p]  No response [q] | **Yes**  1  1  1  1  1  1  1  1  1  1  1  1  1  1  1  1  1 | | | **No**  0  0  0  0  0  0  0  0  0  0  0  0  0  0  0  0  0 | **If response to [p] or [q] is 1, no other 1 responses allowed** |
|  | Approximately how much time went by between when you stopped using the previous method and the day that you recently received your method at the clinic?  ***First choose unit of time, then enter amount.***  ***If participant received new method on same day as stopped using previous method, enter 0 days.*** | *A. Enter unit of time the participant responds in*  Days  Weeks  Months  Years  Not sure  No response | 1  2  3  4  88  99 | | | |  |
|  |  | *B. Enter amount of time, corresponding to unit previously selected.*  *If Not sure or No response, enter 99.*  [__\|__] | | | | |  |

## Section 3: Method Choice

Now let’s talk about [name of method] you recently received at the clinic. I would like to ask you some questions about your choice.

| **No.** | **Question** | **Responses** | **Code** | | | **Skip** |
| --- | --- | --- | --- | --- | --- | --- |
|  | What method did the participant receive? [Code 1 if 1 in Q017] | LNG-IUS  Any other method | 1  2 | | | **🡪Q303** |
|  | Where or how did you hear about the LNG-IUS?  ***Multiple responses possible.***  **Probe:** Anywhere else? | From clinic staff during visit to get method [a]  From clinic staff on another visit to clinic [b]  Referred by other health provider [c]  Community workers [d]  Friend / colleague / family member [e]  Flyers [f]  Radio [g]  Other [h]  Not sure [i]  No response [j] | **Yes**  1  1  1  1  1  1  1  1  1  1 | | **No**  0  0  0  0  0  0  0  0  0  0 | **If response to [i] or [j] is 1, no other 1 responses allowed** |
|  | What are the reasons you chose this method instead of another method?  ***Multiple responses possible.***  **Probe:** Any other reason? | Nobody will know I am using it [a]  Lasts long time [b]  Highly / more effective [c]  Convenient / don’t need to do anything on regular basis [d]  Used before [e]  “Right” for my body [f]  Continue having regular period [g]  Lighter, shorter, or no period [h]  Treats heavy or painful period [i]  Few side effects/Fewer side effects than other methods [j]  Side effects manageable/easier to cope with than other methods [k]  Can get pregnant without problems after using [l]  OK for use while breastfeeding [m]  Low or less hormones [n]  Affordable [o]  Recommended by friend / colleague / family [p]  Recommended by partner [q]  Recommended by provider [r]  Other, specify: _______________ [s]  Not sure [t]  No response [u] | **Yes**  1  1  1  1  1  1  1  1  1  1  1  1  1  1  1  1  1  1  1  1  1 | | **No**  0  0  0  0  0  0  0  0  0  0  0  0  0  0  0  0  0  0  0  0  0 | **If response to [t] or [u] is 1, no other 1 responses allowed** |
|  | Did you already know that you wanted this method before you came to the clinic, or did you decide during your visit? | Already knew  Decided during visit  Not sure  No response | 1  2  88  99 | | |  |
|  | Did anyone influence your decision to use this method? | Made decision on own  Influenced by someone  No response | 1  2  99 | | | **🡪Q307**  **🡪Q307** |
|  | Who influenced you the most to use the method? | Husband / partner  Other family member  Friend / colleague  Provider  Other  No response | 1  2  3  4  5  99 | | | **🡪Q308** |
|  | Does your husband/partner know that you are using this method? | Yes  No  No response | 1  0  99 | | |  |
|  | If the method had not been available during your visit, what method, if any, would you have chosen instead? | Female sterilization  Male sterilization  Copper IUD  LNG-IUS  IUD – unspecified  1-rod implant / Implanon  2-rod implant / Jadelle  Implant – other/unknown  3-month injectable/Depo  2-month injectable/Noristerat  Injectable – other/unknown  Pills  Emergency contraception  Male condoms only  Female condoms only  SDM / CycleBeads  LAM  Diaphragm  Foam / Jelly  Traditional (rhythm, withdrawal, abstinence)  No method  Gone elsewhere for same method  Other, specify: ________________  Not sure  No response | 1  2  3  4  5  6  7  8  9  10  11  12  13  14  15  16  17  18  19  20  21  22  23  88  99 | | |  |
|  | What method did the participant receive? [Code 1 if 1 in Q017] | LNG-IUS  Any other method | 1  2 | | | **🡪Q401** |
|  | Have you ever heard about the LNG-IUS? | Yes  No  Not sure  No response | 1  0  88  99 | | | **🡪Q401**  **🡪Q401**  **🡪Q401** |
|  | Where or how did you hear about the LNG-IUS?  ***Multiple responses possible***  **Probe:** Anywhere else? | From clinic staff during visit to get method [a]  From clinic staff on another visit to clinic [b]  Referred by other health provider [c]  Community workers [d]  Friend / colleague / family member [e]  Flyers [f]  Radio [g]  Other [h]  Not sure [i]  No response [j] | **Yes**  1  1  1  1  1  1  1  1  1 | **No**  0  0  0  0  0  0  0  0  0 | | **If response to [i] or [j] is 1, no other 1 responses allowed** |
|  | Do you think you may be interested in using the LNG-IUS at any time in the future? I am not asking you to commit to using it, I just want to understand what you think about this method. | Yes  No  Not sure  No response | 1  0  88  99 | | | **🡪Q401** |
|  | Why are you not interested in using the LNG-IUS at any time in the future?  ***Multiple responses possible.***  **Probe:** Any other reason? | Fear of insertion procedure [a]  Fear partner will find out or feel strings [b]  Lasts too long [c]  Not effective [d]  Concerns about lighter or shorter periods [e]  No period [f]  Bleeding disturbances (spotting, change in frequency, irregular) [g]  Pain / discomfort with method after inserted [h]  Weight gain [i]  Other side effects [j]  Has hormones [k]  Too expensive [l]  Fear to become infertile / difficult to get pregnant after use [m]  Afraid will travel in body [n]  Other, specify: ______________ [o]  Not sure [p]  No response [q] | **Yes**  1  1  1  1  1  1  1  1  1  1  1  1  1  1  1  1  1 | **No**  0  0  0  0  0  0  0  0  0  0  0  0  0  0  0  0  0 | | **If response to [p] or [q] is 1, no other 1 responses allowed** |

## Section 4: Experiences Getting Method

Now I would like to talk about your experience getting [name of method] at the clinic.

| **No.** | **Question** | **Responses** | **Code** | | | **Skip** |
| --- | --- | --- | --- | --- | --- | --- |
|  | By what means do you most commonly travel from your home to the clinic?  ***If more than one, record the one that is used to complete most of the distance*** | Walk  Bicycle  Motorcycle / scooter  Personal vehicle  Taxi  Bus/public transport  Other, specify: ______________  No response | 1  2  3  4  5  6  7  99 | | |  |
|  | How long does it take to travel from your home to the clinic?  ***Record time for one-way trip.***  ***First choose unit of time, then enter amount.*** | *A. Enter unit of time the participant responds in*  Hours  Minutes  Days  Not sure  No response | 1  2  3  88  99 | | |  |
|  |  | *B. Enter amount of time, corresponding to unit previously selected. If Not sure or No response, enter 99*  [__\|__] | | | |  |
|  | Did you receive your method the first time you went to the clinic to ask for it? | Yes  No  No response | 1  0  99 | | | **🡪Q405**  **🡪Q405** |
|  | Why did you not get it the first time? | Long line / long wait  Method not available  Equipment / supplies not available  Not enough money  Not currently having menses  Needed partner approval  Provider not available  Provider would not give it to me  Just making inquiries the first time  Other  Not sure  No response | 1  2  3  4  5  6  7  8  9  10  88  99 | | |  |
|  | Not including transportation to the clinic, how much did you pay for your method (in Kwacha)? | [_\|_\|_\|_\|_\|_\|_\|_] (Kwacha)  ***If not sure enter 888, if no response enter 999*** | | | |  |
|  | How much did you pay for transportation for the round trip to the clinic (in Kwacha)? | [_\|_\|_\|_\|_\|_\|_] (Kwacha)  ***If not sure enter 888, if no response enter 999*** | | | | **Do not ask if response to Q401=4** |
|  | Did you receive this method within two weeks of having an abortion or a miscarriage? | Yes  No  Not sure  No response | 1  0  88  99 | | |  |
|  | When you obtained your method, did the provider tell you about other methods you could use? | Yes  No  Not sure  No response | 1  0  88  99 | | |  |
|  | Did the provider talk to you about bleeding changes or other side effects that you may experience while using your method? | Yes  No  Not sure  No response | 1  0  88  99 | | | **🡪Q413**  **🡪Q413**  **🡪Q413** |
|  | What possible bleeding changes or side effects did the provider say that you may experience while using your method?  ***Multiple responses possible***  **Probe:** Anything else? | **Bleeding Patterns**  Less bleeding than normal (lighter or shorter) [a]  No bleeding [b]  More bleeding than normal (heavier or longer) [c]  Bleeding disturbances (spotting, change in frequency, irregular) [d]  Less pain during period [e]  **Other side effects**  Vaginal bacterial infections [f]  Yeast infections [g]  Pain during sex [h]  Lack of sexual desire [i]  Pelvic discomfort / pain [j]  Expulsion (IUD coming out on own) [k]  Changes in skin [l]  Headaches [m]  Nausea / vomiting [n]  Abdominal pain [o]  Breast tenderness [p]  Mood changes [q]  Weight gain [r]  Delayed return to fertility [s]  Other, specify: _____________ [t]  Not sure [u]  No response [v] | **Yes**  1  1  1  1  1  1  1  1  1  1  1  1  1  1  1  1  1  1  1  1  1  1 | | **No**  0  0  0  0  0  0  0  0  0  0  0  0  0  0  0  0  0  0  0  0  0  0 | **If response to [u] or [v] is 1, no other 1 responses allowed** |
|  |  |  |  | | |  |
|  |  |  |  | | |  |
|  | Did the provider tell you to come back for follow-up visits for your method? | Yes  No  Only if I have a problem  Not sure  No response | 1  2  3  88  99 | | |  |
|  | What method did the participant receive? [Code 1 if code 4 in Q017] | Injectable  Any other method | 1  2 | | | **🡪Q418** |
|  | How many years did the provider say your method can remain in until it needs to be removed? | Less than 3 years  3 years  4 years  5 years  6-9 years  10-12 years  More than 12 years  Provider didn’t say  Not sure  No response | 1  2  3  4  5  6  7  8  88  99 | | |  |
|  | Did the provider tell you that you can have your method removed at any time you want? | Yes  No  Not sure  No response | 1  0  88  99 | | |  |
|  | Where did the provider say that you could go when you wanted to have your method removed?  ***Multiple responses possible.***  **Probe**: Anywhere else? | At the same clinic where it was inserted ONLY [a]  At a different clinic from where it was inserted ONLY [b]  At any clinic [c]  Provider did not say [d]  Not sure [e]  No response [f] | **Yes**  1  1  1  1  1 | **No**  0  0  0  0  0 | | **If response to [e] or [f] is 1, no other 1 responses allowed**  **All**  **🡪Q419** |
|  | How long did the provider tell you the injection would protect you against pregnancy? | 3 months / 13 weeks  Any other duration  Provider didn’t say  Not sure  No response | 1  2  3  88  99 | | |  |
|  | Do you feel that you had enough privacy when the provider gave you your method? | Yes  No  No response | 1  0  99 | | |  |
|  |  |  |  | | |  |
|  |  |  |  | | |  |
|  | Did you have any problems when the provider gave you your method? | Yes  No  Not sure  No response | 1  0  88  99 | | | **🡪Q425**  **🡪Q425**  **🡪Q425** |
|  | What problem(s) did you have?  ***Multiple responses possible***  **Probe:** Anything else? | Temporary pain at time of insertion [a]  Discomfort / pain that lasted a few days but then went away [b]  Continuing pain that hasn’t gone away [c]  Cramping [d]  Infection / swelling [e]  Scarring [f]  Other [g]  No response [h] | **Yes**  1  1  1  1  1  1  1  1 | **No**  0  0  0  0  0  0  0  0 | | **If response to [h] is 1, no other 1 responses allowed** |
|  |  |  |  | | |  |
|  | Overall, can you rate your experience with your method so far? Would you say that you are…(READ RESPONSES) | Very satisfied  Somewhat satisfied  Neither satisfied nor dissatisfied  Somewhat dissatisfied  Very dissatisfied  ***Do not read****:* No response | 1  2  3  4  5  99 | | |  |
|  | How long do you plan to keep using your method? | Less than 1 year  1-2 years  3-5 years  More than 5 years  Undecided  Until no longer effective  Until I can no longer get pregnant  Other  No response | 1  2  3  4  5  6  7  8  9 | | |  |

## Section 5: Wealth Quintiles

Thank you. Before our interview ends I would like to ask you a few questions about your household to better understand the general background of women using contraceptives like you. A household consists of the people who live together in the same dwelling unit and who eat food from the same pot. In some cases, one household may live in multiple dwelling units like a compound house.

| **No.** | **Question** | **Responses** | **Code** | | **Skip** |
| --- | --- | --- | --- | --- | --- |
|  | Does your household have: (READ ALOUD, ONE BY ONE) | Electricity [a]  A television [b]  A refrigerator [c]  A sofa [d]  A clock [e]  A fan [f]  A VCR/DVD [g] | **Yes**  1  1  1  1  1  1  1 | **No**  0  0  0  0  0  0  0 |  |
|  | What is the main material of the roof of your household’s house? | No roof  Thatch / palm leaf  Iron sheets  Other, specify: _______________  No response | 1  2  3  4  99 | |  |
|  | What is the main material of your household’s floor? | Earth / sand  Dung  Concrete cement  Other, specify: _______________  No response | 1  2  3  4  99 | |  |
|  | What type of fuel does your household mainly use for cooking? | Electricity  Charcoal  Other, specify: _______________  No response | 1  2  3  99 | |  |
|  | Does any member of this household have a bank account? | Yes  No  No response | 1  2  99 | |  |

Thank you for taking the time to speak with me today. Do you have any questions?

The next surveys will be shorter and only ask about your experiences with your method. We will not ask you questions about yourself or your household again.

If your address or phone number change before we speak again, please tell me or call or text me, **[RA name]**, at **[phone number]** so that I can reach you again. [Give slip with information]
